# Red ginseng abrogates oxidative stress via mitochondria protection mediated by LKB1-AMPK pathway

**DOI:** 10.1186/1472-6882-13-64

**Published:** 2013-03-18

**Authors:** Guang-Zhi Dong, Eun Jeong Jang, Seung Ho Kang, Il Je Cho, Sun-Dong Park, Sang Chan Kim, Young Woo Kim

**Affiliations:** 1 Medical research center for Globalization of Herbal Formulation, College of Oriental Medicine, Daegu Haany University, Daegu 706-828, Korea; 2 Sunlin University, Pohang, Kyungsangbuk-do 791-712, Korea

**Keywords:** Arachidonic acid, Red ginseng, AMPK, Oxidative stress, Mitochondria

## Abstract

**Background:**

Korean ginseng (*Panax ginseng* C.A. Meyer) has been used as a botanical medicine throughout the history of Asian traditional Oriental medicine. Formulated red ginseng (one form of Korean ginseng) has been shown to have antioxidant and chemopreventive effects.

**Methods:**

This study investigated the cytoprotective effects and mechanism of action of Korean red ginseng extract (RGE) against severe ROS production and mitochondrial impairment in a cytotoxic cell model induced by AA + iron.

**Results:**

RGE protected HepG2 cells from AA + iron-induced cytotoxicity by preventing the induction of mitochondrial dysfunction and apoptosis. Moreover, AA + iron-induced production of ROS and reduction of cellular GSH content (an important cellular defense mechanism) were remarkably attenuated by treatment with RGE. At the molecular level, treatment with RGE activated LKB1-dependent AMP-activated protein kinase (AMPK), which in turn led to increased cell survival. The AMPK pathway was confirmed to play an essential role as the effects of RGE on mitochondrial membrane potential were reversed upon treatment with compound C, an AMPK inhibitor.

**Conclusions:**

Our results demonstrate that RGE has the ability to protect cells from AA + iron-induced ROS production and mitochondrial impairment through AMPK activation.

## Background

During oxidation of fatty acids and phospholipids, phospholipase A_2_ triggers the release of arachidonic acid (AA), a ω-6 polyunsaturated fatty acid [1,2]. As a biologically active pro-inflammatory mediator, AA can induce apoptosis through its effects on mitochondria (e.g. calcium uptake into mitochondria, or production of ceramide) [1,2]. Furthermore, in the presence of iron, which is a catalyst of auto-oxidation, AA stimulates cells to produce excess ROS, resulting in induction of mitochondrial dysfunction [3-7]. AMP-activated protein kinase (AMPK, an important molecule sensing cellular energy status) is activated to reserve cellular energy content, and it plays a function in determining cell survival or death in pathological progression [7,8]. This crucial role is supported by increases in cell survival upon treatment with the AMPK activators metformin and 5-aminoimidazole-4-carboxamide-1-β-D-ribofuranoside (AICAR) [9,10]. Moreover, a line of agents protecting cells has been shown to inhibit radical-induced stress through AMPK activation as well as induction of antioxidant enzymes [11,12].

Korean ginseng (*Panax ginseng* C.A. Meyer) is one of the oldest and most frequently used botanicals in the history of traditional Oriental medicine. Korean ginseng extract is recommended for its life-enhancing properties as well as promotion of energy and longevity. Studies have shown that ginseng attenuates free radical-induced oxidative damage [13,14], prevents carcinogenesis induced by toxicants [15], and possesses immunostimulating, anti-tumorigenic, and chemopreventive effects [16-18]. These numerous cytoprotective and chemoprotective properties attributed to ginseng might be explained in part by its ability to ameliorate oxidative or nitrosative stress [19]. Korean red ginseng is one form of Korean ginseng that is marinated in an herbal brew (i.e. heating *Panax ginseng* either by sun-drying or steaming), resulting in the root becoming extremely fragile. It has been shown that red ginseng inhibits oxidative cell death through Nrf2 activation and protects smokers from oxidative DNA damage [20,21]. Although the biological effects of red ginseng have been well studied, it is not yet clear whether or not its cytoprotective effects against mitochondrial impairment are induced by AA + iron.

In view of the numerous beneficial effects of red ginseng as well as the importance of AMPK in the protection of mitochondria, this study investigated whether or not Korean red ginseng extract (RGE) is capable of protecting mitochondria against the severe oxidative stress induced by AA + iron and, if so, whether or not this extract has the ability to prevent apoptosis. Our work demonstrates that RGE protects cells against severe oxidative burst by inhibiting mitochondrial impairment and ROS production through AMPK activation.

## Methods

### Reagent

RGE was provided by Korea Tobacco & Ginseng Corporation (Daejeon, Korea) [22]. AA and compound C were purchased from Calbiochem (San Diego, CA). Anti-procaspase-3, anti-phospho-acetyl-CoA carboxylase (ACC), anti-PARP, anti-phospho-LKB1 and anti-phospho-AMPK antibodies were obtained from Cell Signaling Technology (Beverly, MA). Anti-AMPK, anti-ACC and anti-LKB1 antibodies were purchased from Santa Cruz Biotechnology (Santa Cruz, CA). Horseradish peroxidase-conjugated goat anti-rabbit, rabbit anti-goat, and goat anti-mouse IgGs were obtained from Zymed Laboratories (San Francisco, CA). Ferric nitrate, nitrilotriacetic acid [9], 3-(4,5-dimethylthiazol-2-yl)-2,5-diphenyl-tetrazolium bromide (MTT), rhodamine 123, 2^′^,7^′^-Dichlorofluorescein diacetate (DCFH-DA), anti-β-actin antibody, and other reagents were purchased from Sigma (St. Louis, MO). The solution of iron-NTA complex was prepared as described previously [7].

### Cell culture

HepG2 (human), H4IIE (rat), and AML12 (mouse) hepatocyte-derived cell lines were purchased from ATCC (Rockville, MD). Cells were incubated in Eagle’s minimum essential medium without 10% FBS for 12 h. Then, cells were incubated with 10 μM AA for 12 h, followed by exposure to 5 μM iron after washing with PBS. To assess the effects of RGE, the cells were treated with RGE for 1 h prior to the incubation with AA at the indicated doses [12].

### MTT assay

The MTT assay was performed as previously described [12]. Briefly, HepG2 cells were plated at a density of 1 × 10^5^ cells per well in a 48-well plate. After treatment, viable cells were stained with 0.25 mg/ml MTT for 2 h. The media was then removed, and formazan crystals produced in the wells were dissolved with the addition of 200 μl dimethylsulfoxide. Absorbance at 540 nm was measured using an ELISA microplate reader (Tecan, Research Triangle Park, NC). Cell viability was defined relative to untreated control [i.e. viability (% control) = 100 × (absorbance of treated sample)/ (absorbance of control)].

### Terminal deoxynucleotidyl transferase dUTP nick end labeling (TUNEL) assay

The TUNEL assay was performed using the DeadEnd™ Colorimetric TUNEL System, according to the manufacturer’s instruction [23]. HepG2 cells were fixed with 10% buffered formalin in PBS at room temperature for 30 min and were permeabilized with 0.2% Triton X-100 for 5 min. After washing with PBS, each sample was incubated with biotinylated nucleotide and terminal deoxynucleotidyltransferase in 100 μl equilibration buffer at 37°C for 1 h. The reaction was stopped by immersing the samples in 2× saline sodium citrate buffer for 15 min. Endogenous peroxidases were blocked by immersing the samples in 0.3% H_2_O_2_ for 5 min. The samples were treated with 100 μl of horseradish peroxidase-labeled streptavidin solution (1:500) and were incubated for 30 min. Finally, the samples were developed using the chromogen, H_2_O_2_ and diaminobenzidine for 10 min. The samples were washed and examined under light microscope (200×). The counting of TUNEL-positive cells was repeated three times, and the percentage from each counting was calculated.

### Immunoblot analysis

Cell lysates and Immunoblot analysis were performed according to previously published methods [23]. Protein bands of interest were developed using an ECL chemiluminescence system (Amersham, Buckinghamshire, UK). Equal protein loading was verified by immunoblotting for β-actin.

### Measurement of H_2_O_2_ production

DCFH oxidation was determined using a FACS flow cytometer (Partec, Münster, Germany). DCFH-DA is a cell-permeable non-fluorescent probe that is cleaved by intracellular esterases and is turned into the fluorescent DCF upon reaction with H_2_O_2_[23]. The level of H_2_O_2_ generation was determined by the concomitant increase in DCF fluorescence. After treatment, cells were stained with 10 μM DCFH-DA for 1 h at 37°C. Fluorescence intensity in the cells was measured using FACS. In each analysis, 10,000 events were recorded.

### Determination of reduced GSH content

Reduced GSH in the cells was quantified using a commercial GSH determination kit (Oxis International, Portland, OR) [12]. Briefly, the GSH-400 method was a two-step chemical reaction. The first step led to the formation of substitution products (thioethers) between 4-chloro-1-methyl-7-trifluromethyl-quinolinum methylsulfate and all mercaptans present in the sample. The second step included β-elimination reaction under alkaline conditions. This reaction was mediated by 30% NaOH which specifically transformed the substituted product (thioether) obtained with GSH into a chromophoric thione.

### Flow cytometric analysis of mitochondrial membrane potential (MMP)

MMP was measured with rhodamine 123, a membrane-permeable cationic fluorescent dye [12]. The cells were treated as specified, stained with 0.05 μg/ml rhodamine 123 for 1 h, and harvested by trysinization. The change in MMP was monitored using a FACS flow cytometer (Partec, Münster, Germany). In each analysis, 10,000 events were recorded.

### Data analysis

One way analysis of variance procedures were used to assess significant differences among treatment groups. For each significant treatment effect, the Newman-Keuls test was utilized to compare multiple group means.

## Results

### Inhibition of AA + iron-induced hepatocyte death

**Figure 1:**
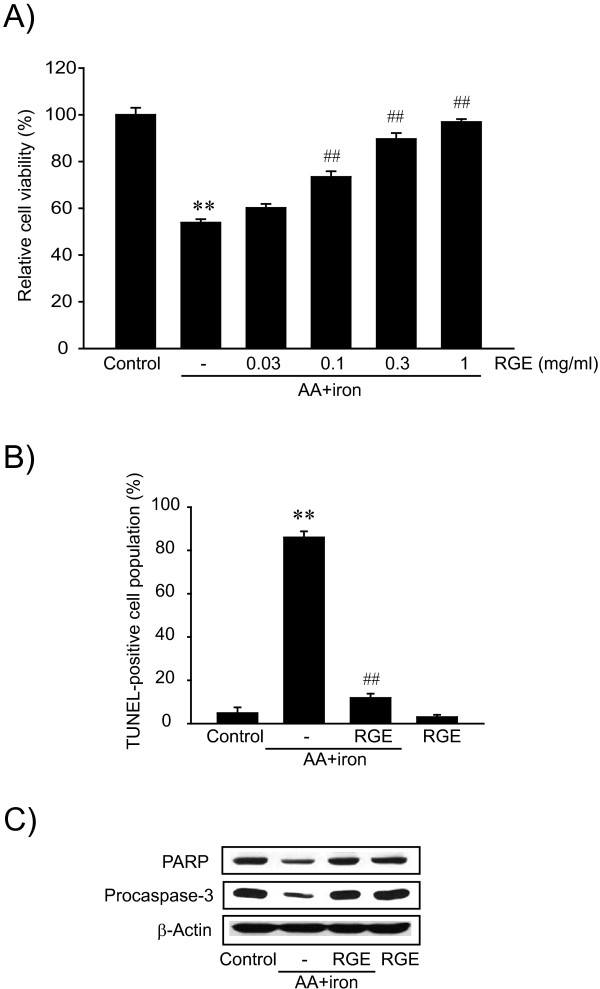
**The effect of Korean red ginseng extract (RGE) on hepatocyte viability. A**) The effect of RGE on hepatocyte viability. HepG2 cells were incubated with 10 μM arachidonic acid (AA) for 12 h and then were treated with 5 μM iron for 6 h. Cell viability was assessed by the MTT assay. Data represent the mean ± S.E.M. of five replicates (treatment mean significantly different from vehicle-treated control, ^**^p < 0.01; treatment mean significantly different from AA + iron, ^##^p < 0.01). **B**) TUNEL assay. HepG2 cells were treated with 1 mg/ml RGE for 1 h and were continuously incubated with 10 μM AA for 12 h, followed by exposure to 5 μM iron for 6 h. The percentage of TUNEL-positive cells was quantified. Data represent the mean ± S.E.M. of three separate experiments (treatment mean significantly different from vehicle-treated control, ^**^p < 0.01; treatment mean significantly different from AA + iron, ^##^p < 0.01). **C**) Immunoblottings for the proteins associated with apoptosis. Immunoblot analyses were performed on the lysates of HepG2 cells that had been incubated with 1 mg/ml RGE for 1 h, continuously treated with 10 μM AA for 12 h, and then exposed to 5 μM iron for 3 h. Equal protein loading was verified by β-actin immunoblotting. Results were confirmed by repeated experiments.

AA + iron-induced cytotoxicity model is an effective experimental model for screening drugs for liver disease [7]. To determine whether or not RGE protects liver cells from AA + iron-induced injury, HepG2 cell viability was measured by MTT assay after treatment with different doses of RGE. Treatment with AA + iron significantly reduced cell viability compared with the control group as shown in Figure 1A. However, RGE treatment inhibited AA + iron treatment-induced cell death in a dose-dependent manner, and cell viability was completely recovered by treatment with 1 mg/ml of RGE (Figure 1A). To further investigate the cytoprotective effects of RGE on AA + iron-induced liver cell injury, TUNEL assay was performed at a dose of 1 mg/ml. Treatment with 1 mg/ml of RGE alone did not induce hepato-cytotoxicity, whereas the same dose (1 mg/ml) of RGE significantly reduced AA + iron-induced cell death (Figure 1B). To confirm the cytoprotective effects of RGE on AA + iron-induced cell death, the levels of PARP and procaspase-3 were measured by immunoblot analysis. Treatment with AA + iron induced cleavage of PARP and procaspase-3, resulting in cell death. In contrast, decreases in the levels of PARP and procaspase-3 induced by AA + iron were inhibited by treatment with RGE (Figure 1C). These results indicate that RGE has cytoprotective effects against apoptosis in hepatocytes induced by AA + iron.

### Inhibition of AA + iron-induced ROS generation

**Figure 2:**
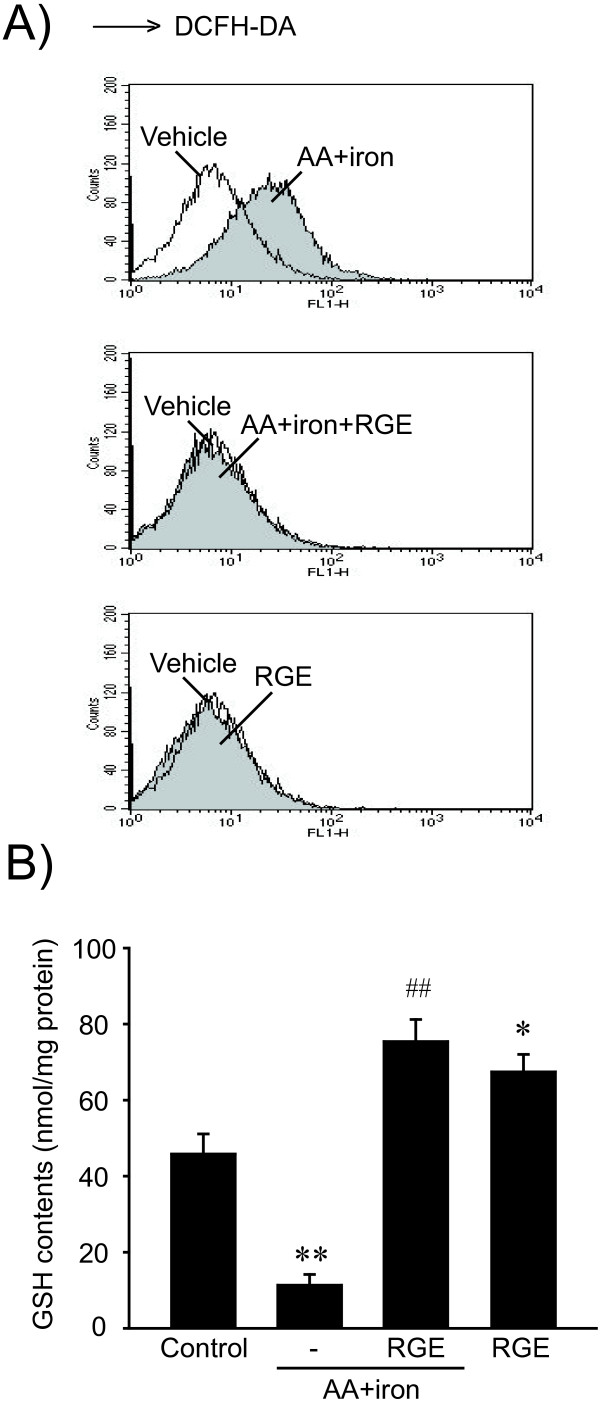
**The cellular antioxidant effect of RGE. A**) Cellular H_2_O_2_ production. H_2_O_2_ production was monitored by measuring dichlorofluorescein (DCF) fluorescence. HepG2 cells were incubated with 1 mg/ml RGE for 1 h, followed by incubation with AA (12 h) and iron (1 h). RGE treatment attenuated AA + iron-induced ROS production. **B**) Cellular GSH content. The GSH content was assessed in cells that had been treated as described in the legend to Figure 1C. Data represent the mean ± S.E.M. of three separate experiments. The statistical significance of differences between treatments and either the vehicle-treated control (^*^p < 0.05, ^**^p < 0.01) or cells treated with AA + iron (^##^*p* < 0.01) was determined.

To investigate the mechanism underlying the protective effects of RGE on AA + iron-induced liver cell death, ROS generation was measured by FACS with or without RGE treatment. There was no ROS generation in RGE alone-treated cells comparable to control cells. AA + iron treatment significantly induced ROS generation, whereas RGE treatment completely inhibited ROS production (Figure 2A). To further investigate the anti-oxidative effects of RGE on AA + iron-treated cells, GSH was measured by the colorimetric method. The intracellular concentration of GSH in HepG2 cells was reduced by treatment with AA + iron (Figure 2B). Treatment with RGE increased the intracellular concentration of GSH and inhibited the AA + iron-induced reduction of GSH. Taken together, these data indicate that RGE inhibits AA + iron-induced ROS generation and GSH reduction.

### Inhibition of MMP dysfunction

**Figure 3:**
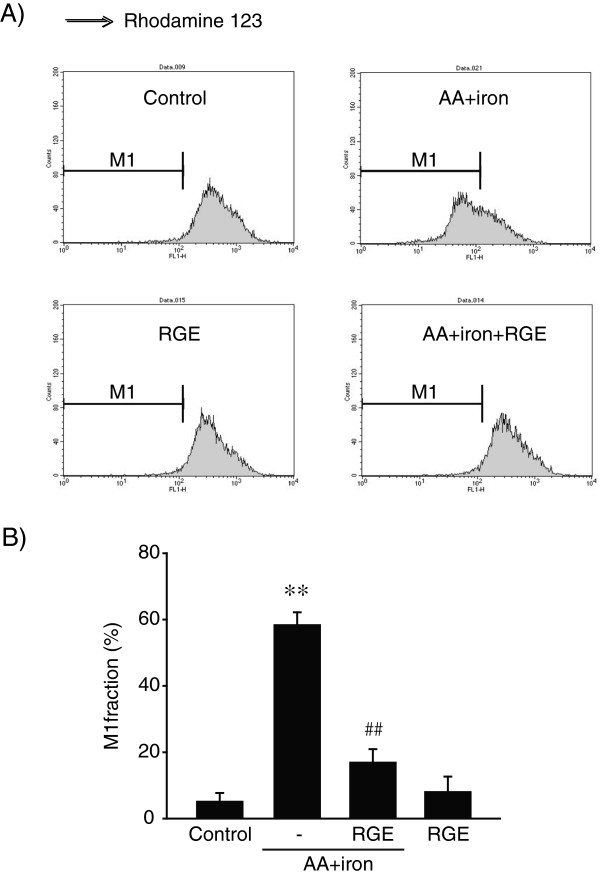
**Abrogation of mitochondrial dysfunction by RGE. A**) Mitochondrial membrane permeability (MMP). HepG2 cells were treated with 1 mg/ml RGE for 1 h, followed by incubation with AA (12 h) and iron (1 h). The cells were harvested after rhodamine 123 staining. Treatment of cells with AA + iron increased the subpopulation of M1 fraction (low rhodamine 123 fluorescence), as indicated by the left shift of the population. **B**) Relative MMP. Data represent the mean ± S.E.M. of three separate experiments. The statistical significance of differences between treatments and either the vehicle-treated control (^**^p < 0.01) or cells treated with AA + iron (^#^*p* < 0.05, ^##^*p* < 0.01) was determined.

Next, we determined whether or not AA + iron-induced liver cell death is mediated by mitochondrial dysfunction. We measured fluorescence intensity in HepG2 cells stained with rhodamine 123 by FACS. MMP was not altered by treatment with RGE alone compared with the control group (Figure 3A). The number of rhodamine 123-negative cells increased AA + iron treatment, whereas it was significantly reduced by RGE co-treatment (Figure 3B). These results indicate that RGE prohibits AA + iron-induced ROS generation and dysfunction of MMP to protect liver cells.

### Activation of AMPK-ACC pathway via phosphorylation of LKB1

**Figure 4:**
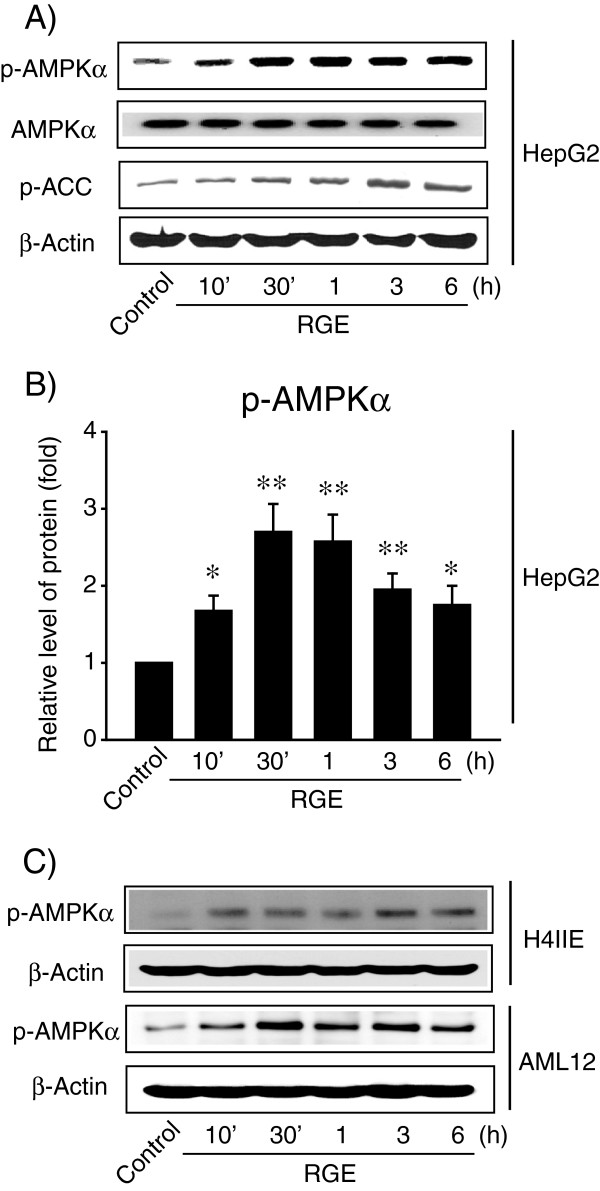
**The activation of AMPK by RGE. A**) AMPK activation. Immunoblot analyses were performed on lysates of HepG2 cells that had been treated with RGE for the indicated time period. **B**) Relative protein level of AMPKα phosphorylation (p-AMPKα). Results were confirmed by three experiments. Data represent the mean ± S.E.M. (treatment mean significantly different from vehicle-treated control, ^*^p < 0.05, ^**^p < 0.01). **C**) AMPK activation by RGE in H4IIE and AML12 cell lines.

To further investigate the mechanism of RGE during hepatocyte protection, the AMPK pathway was analyzed by immunoblot analysis. The phosphorylation levels of AMPK and ACC increased upon RGE treatment, and protein levels reached their maximums at 0.5-1 h and 1-3 h, respectively (Figure 4A and B). AMPK and ACC were also phosphorylated upon RGE treatment in both H4IIE and AML12 immortalized hepatocyte cell lines (Figure 4C).

**Figure 5:**
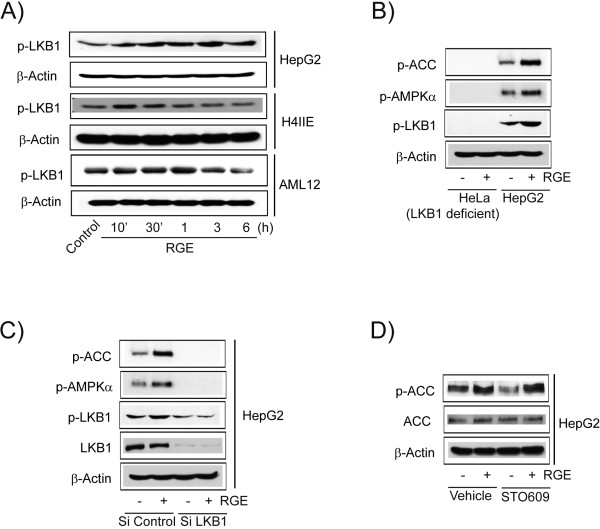
**The activation of AMPK by LKB1. A**) LKB1 activation. Immunoblot analyses were performed on lysates of HepG2, H4IIE and AML12 cells that had been treated with RGE for the indicated time period. **B**) Phosphorylation of AMPKα by LKB1. Immunoblot analyses were performed on lysates of LKB1 null HeLa cell and LKB1 wild-type HepG2 cells following treatment of RGE for 30 mins. Results were confirmed by repeated experiments. **C**) Reduction of AMPKα phosphorylation by knock-down LKB1. **D**) The effect of CaMKK-β inhibitor on the activation of AMPK by RGE. After treatment with 1 μg/ml STO-609, HepG2 cells were continuously incubated with RGE.

LKB1, the upstream kinase of AMPK, was also phosphorylated by RGE treatment in HepG2, H4IIE, and AML12 cell lines (Figure 5A). Phosphorylation of AMPK and ACC was not detectable in LKB1-null HeLa cells (Figure 5B). Furthermore, phosphorylation of AMPK and ACC was decreased by knock-down of LKB1 in RGE-treated HepG2 cells (Figure 5C). Additionally, STO609 (1 μg/ml), an inhibitor of calcium/calmodulin-dependent kinase kinase (CaMKK) β, another upstream kinase of AMPKα, had no effect in reversing RGE-induced AMPKα phosphorylation (Figure 5D). These data indicate that RGE treatment activates the AMPK-ACC pathway in hepatocytes via activation of LKB1.

### Inhibition of AA + iron-induced stress via AMPK pathway

**Figure 6:**
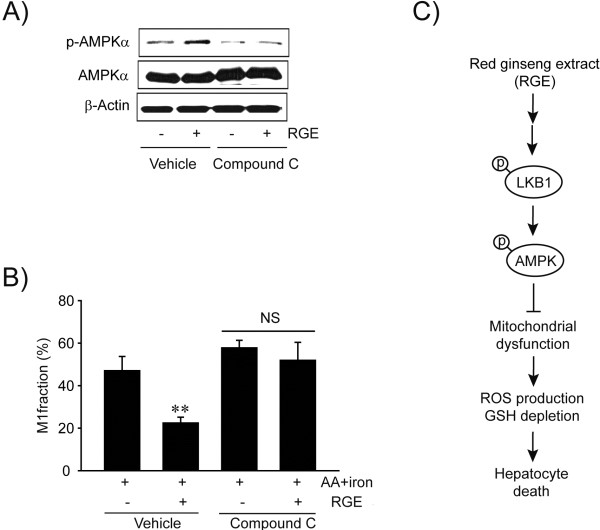
**The role of AMPK activation in protecting mitochondrial function. A**) Inhibition of RGE-induced AMPKα phosphorylation by compound C treatment. Cells were incubated with RGE for 30 min following treatment of 5 μM compound C for 30 min. Results were confirmed by repeated experiments. **B**) Reversal of the effect of RGE on MMP by compound C. After treatment with 5 μM compound C for 30 min, cells were incubated with RGE and/or iron + AA. The subpopulation of M1 fraction was analyzed as described in the legend to Figure 3A. Data represent the mean ± S.E.M. of three replicates (treatment mean significantly different from iron + AA, ^**^p < 0.01). NS, not significant. **C**) A schematic diagram illustrating the proposed mechanism by which RGE protects hepatocytes against AA + iron-induced oxidative stress.

To determine the involvement of AMPK in RGE-induced protection of hepatocytes, MMP was measured after treatment with Compound C, an AMPK inhibitor (Figure 6A). There was no protective effect of RGE against AA + iron-induced mitochondrial dysfunction in AMPK inhibitor-treated cells (Figure 6B), verifying that AMPK is the key protein protecting liver cells upon RGE treatment. All of these data indicate that RGE activates the AMPK pathway to protect hepatocytes against AA + iron (Figure 6C).

## Discussion

Korean red ginseng is frequently used as a crude substance in traditional Oriental medicine and is also a well-known, highly used raw medicinal material. RGE have been reported to exhibit various biological activities, including anti-inflammatory and antitumor effects [16-18]. In this study, we report that RGE has cytoprotective effects against AA + iron-induced oxidative burst, as confirmed by inhibition of apoptosis, ROS production, and mitochondrial dysfunction, which were comparable to the efficacies of other known antioxidants (e.g., resveratrol and some flavonoids) [7,12,23,24]. Our results provide evidence that RGE may be beneficial for treatment of liver diseases by protecting cells from radical stress-induced damage.

AA, a representative pro-inflammatory fatty acid derived from cell membranes, stimulates ROS generation, thereby inducing lipid peroxidation. AA is an important mediator of the pathophysiological processes of various diseases, although the role of AA in responding to toxic stress remains controversial. In most cases, AA promotes cellular ROS production and induces decreases in mitochondrial respiratory activity, and ROS generated by metabolism of AA contributes to the process of tissue damage [25,26]. In addition, AA releases Ca^2+^ from intracellular stores and increases mitochondrial uptake of Ca^2+^, which may cause apoptosis [27]. In other cases, prostaglandins, the main byproducts of AA, may be responsible for the protection of some tissues [28,29]. Nevertheless, AA-stimulated oxidative stress has been shown to have a direct effect on mitochondria [1,2].

Iron accumulation in specific tissues (e.g. liver) is commonly associated with oxidative and inflammatory damage, including metabolic disease and cancer [3,30], which enhances oxidant production, lipid peroxidation, protein oxidation, and DNA damage. Since iron is a catalyst of auto-oxidation, the combination of AA and iron increases radical stress and cell death in a synergistic manner [7,12,31]. Moreover, HepG2 cells were used to apply the well-established culture conditions of synergism to this model. In fact, a series of cytoprotective and important agents have been evaluated using this model [7,12,24,32]. This cell line was employed to comparatively evaluate the protective effects of RGE in cells and mitochondria. To determine the effects of RGE on oxidative stress, we employed an *in vitro* approach using a combination treatment with AA and iron to HepG2 cells.

AMPK (an intracellular sensor of energy status) serves as a crucial regulator of cell survival or death in response to pathological stress (e.g., oxidative stress, endoplasmic reticulum stress, and osmotic stress) [8,33]. This important function of AMPK is supported by the finding that cell viability is increased by treatment with AMPK activators, including AICAR or resveratrol [12,24]. Moreover, a series of beneficial compounds have shown the ability to protect mitochondria, thereby inhibiting ROS production through activation of AMPK (e.g., oltipraz, resveratrol, isoliquiritigenin, and sauchinone) [7,12,23,24].

In the present study, RGE activated AMPK in hepatocytes. Moreover, AMPK inhibition induced by compound C also prevented the ability of RGE to increase dysfunction of MMP, suggesting that AMPK indeed inhibits AA + iron-induced oxidative stress. In mammalian cells, LKB1 and CaMKKβ are the major upstream kinases of AMPK [34,35]. RGE phosphorylation of AMPK was inhibited by LKB1 knock-down but not by treatment with CaMMK inhibitor. Overall, it appears that AMPK activation induced by RGE may protect hepatocytes against AA + iron-induced oxidative stress. However, LKB1-AMPK might not be a direct target of RGE. Protein kinase C-ζ or protein kinase A are the kinases that phosphorylate LKB1, an upstream kinase of AMPK [36]. The pharmacological upstream target of RGE remains to be confirmed.

## Conclusions

Our results demonstrate that RGE exerts cytoprotective effects by increasing antioxidant capacity and recovery of mitochondrial function, which may be associated with AMPK activation. The present results may be informative in elucidating the action mechanism and efficacy of RGE in hepatocyte protection as well as in determining its potential in treating various diseases related with oxidative stress.

## Competing interests

The authors declare that they have no competing interests.

## Authors’ contributions

GZD: acquisition of data; analysis and interpretation of data; statistical analysis; drafting of the manuscript. EJJ: acquisition of data; analysis and interpretation of data. SHK: acquisition of data; analysis and interpretation of data. IJC: obtained funding; administrative support. SDP: analysis and interpretation of data; review of the manuscript. SCK: analysis and interpretation of data; obtained funding; administrative support; study supervision. YWK: acquisition of data; analysis and interpretation of data; drafting of the manuscript; statistical analysis; obtained funding; study supervision. All authors read and approved the final manuscript.

## Pre-publication history

The pre-publication history for this paper can be accessed here:

http://www.biomedcentral.com/1472-6882/13/64/prepub
